# 2-(4-Oxo-3-phenyl-1,3-thia­zolidin-2-yl­idene)propanedi­nitrile

**DOI:** 10.1107/S1600536813018308

**Published:** 2013-07-24

**Authors:** Mehmet Akkurt, Ahmed M. M. El-Saghier, Sabry H. H. Younes, Peter N. Horton, Mustafa R. Albayati

**Affiliations:** a Department of Physics, Faculty of Sciences, Erciyes University, 38039 Kayseri, Turkey; b Chemistry Department, Faculty of Science, Sohag University, 82524-Sohag, Egypt; c School of Chemistry, University of Southampton, Highfield, Southampton SO17 1BJ, England; d Kirkuk University, College of Science, Department of Chemistry, Kirkuk, Iraq

## Abstract

In the title compound, C_12_H_7_N_3_OS, the five-membered 1,3-thia­zolidine ring is nearly planar [maximum deviation = 0.032 (2) Å] and makes a dihedral angle of 84.14 (9)° with the phenyl ring. In the crystal, mol­ecules are linked by C—H⋯N hydrogen bonds into infinite chains along [-101]. C—H⋯π inter­actions contribute to the arrangement of the mol­ecules into layers parallel to (101).

## Related literature

For the diverse biological applications of thia­zolidinone-containing compounds, see, for example: Bouzroura *et al.* (2010 ▶); Abhinit *et al.* (2009 ▶); Naeem *et al.* (2009 ▶); Sharma *et al.* (2009 ▶); Mistry & Desai (2004 ▶); Ramalakshmi *et al.* (2009 ▶); Turgut *et al.* (2007 ▶). For the synthesis of similar compounds, see: Farhat *et al.* (2007 ▶). For similar structures, see: Pomés Hernández *et al.* (1996 ▶).
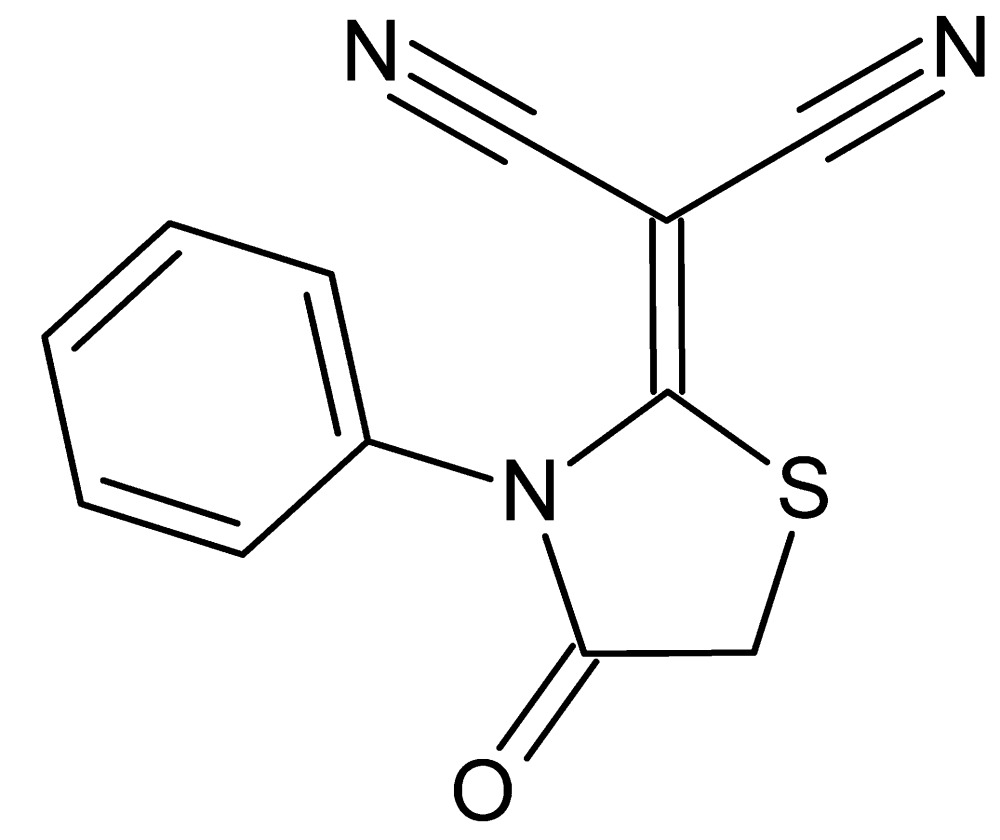


## Experimental

### 

#### Crystal data

C_12_H_7_N_3_OS
*M* *_r_* = 241.28
Monoclinic, 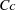

*a* = 16.979 (9) Å
*b* = 9.407 (5) Å
*c* = 7.034 (4) Å
β = 103.927 (11)°
*V* = 1090.5 (10) Å^3^
*Z* = 4
Mo *K*α radiation
μ = 0.28 mm^−1^
*T* = 100 K
0.24 × 0.12 × 0.04 mm

#### Data collection

Rigaku AFC12 (Right) diffractometer
Absorption correction: multi-scan (*CrystalClear-SM Expert*; Rigaku, 2012 ▶) *T*_min_ = 0.944, *T*_max_ = 1.000
3632 measured reflections
1986 independent reflections
1955 reflections with *I* > 2σ(*I*)
*R*_int_ = 0.015

#### Refinement

*R*[*F* ^2^ > 2σ(*F* ^2^)] = 0.022
*wR*(*F* ^2^) = 0.061
*S* = 1.08
1986 reflections
154 parameters
2 restraints
H-atom parameters constrained
Δρ_max_ = 0.19 e Å^−3^
Δρ_min_ = −0.18 e Å^−3^
Absolute structure: Flack *x* parameter determined using 718 quotients [(I^+^)−(I^−^)]/[(I^+^)+(I^−^)] (Parsons & Flack, 2004 ▶)
Flack parameter: 0.03 (3)

### 

Data collection: *CrystalClear-SM Expert* (Rigaku, 2012 ▶); cell refinement: *CrystalClear-SM Expert*; data reduction: *CrystalClear-SM Expert*; program(s) used to solve structure: *SHELXS97* (Sheldrick, 2008 ▶); program(s) used to refine structure: *SHELXL97* (Sheldrick, 2008 ▶); molecular graphics: *ORTEP-3 for Windows* (Farrugia, 2012 ▶); software used to prepare material for publication: *WinGX* (Farrugia, 2012 ▶) and *PLATON* (Spek, 2009 ▶).

## Supplementary Material

Crystal structure: contains datablock(s) global, I. DOI: 10.1107/S1600536813018308/hg5329sup1.cif

Structure factors: contains datablock(s) I. DOI: 10.1107/S1600536813018308/hg5329Isup2.hkl

Supplementary material file. DOI: 10.1107/S1600536813018308/hg5329Isup3.cml

Additional supplementary materials:  crystallographic information; 3D view; checkCIF report

## Figures and Tables

**Table 1:** Hydrogen-bond geometry (Å, °) *Cg*2 is the centroid of the C7–C12 phenyl ring.

*D*—H⋯*A*	*D*—H	H⋯*A*	*D*⋯*A*	*D*—H⋯*A*
C10—H10⋯N3^i^	0.95	2.58	3.479 (4)	157
C8—H8⋯*Cg*2^ii^	0.95	2.96	3.610 (3)	127

Symmetry codes: (i) 
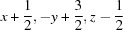
; (ii) 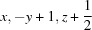.

**Fig. 1.**
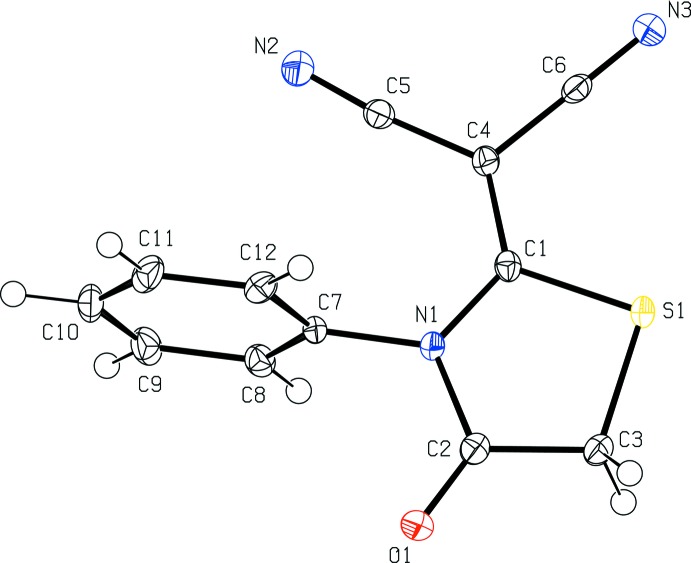
The molecular structure of the title compound with the atom numbering scheme. Displacement ellipsoids for non-H atoms are drawn at the 50% probability level.

**Fig. 2.**
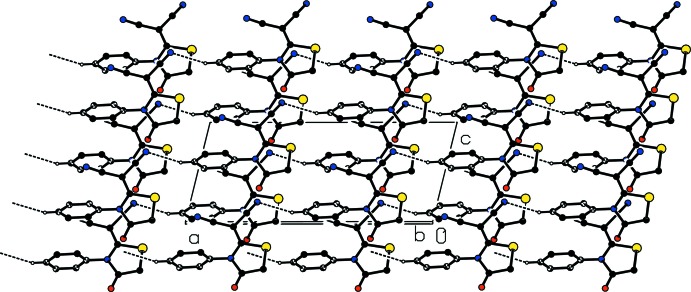
A view along the b axis of the packing diagram of (I) showing hydrogen bonds as dashed lines. H atoms not involved in hydrogen bonds have been omitted for clarity.

## supplementary crystallographic information

### Comment

Compounds containg thiazolidinone ring system have been found to possess a broad spectrum of biological activities (Abhinit *et al.*, 2009). 4-Thiazolidinones is a core structure in various synthetic compounds and an important scaffold known to be associated with several biological activities such as, antitubercular (Naeem *et al.*, 2009), anti bacterial (Sharma *et al.*, 2009), anti-inflammatory (Turgut *et al.*, 2007), anti-mycobacterial (Bouzroura *et al.*, 2010), anti convulsant (Mistry & Desai, 2004), and anti cancer (Ramalakshmi *et al.*, 2009). As such we have synthesized in our lab series of thiazolidinone derivatives and herein we report the crystal structure of the title compound (I).

In (I), (Fig. 1), the five-membered 1,3-thiazolidine ring (S1/N1/C1–C3) is nearly planar with maximum deviations of 0.031 (1) Å for S1 and -0.032 (2) Å for C3. The dihedral angle between the 1,3-thiazolidine ring and phenyl rings (S1/N1/C1–C3 and C7–C12) is 84.14 (9)°. In (I), the C4–C5–N2, C4–C6–N3 and C5–C4–C6 angles are 174.4 (2), 179.4 (2) and 115.80 (18)°, respectively. The N1–C1–C4–C5 and N1–C1–C4–C6 torsion angles are -0.5 (3) and 177.33 (18)°, respectively. The values of the geometric parameters are normal and are comparable to those observed in similar compounds (Pomés Hernández *et al.*, 1996).

In the crystal structure, C—H···N hydrogen bonds (Table 1, Fig. 2) link the molecules to each other into infinite chains along the [-101] direction. The molecules are arranged into layers parallel to (101) through C—H···π interactions between the C(8)H8 atoms and the centroids of the phenyl rings of neighbouring molecules.

### Experimental

The title compound has been prepared according to the our reported method (Farhat *et al.*, 2007). Pale brown mono-crystals suitable for X-ray diffractions were grown up by slow evaporation of an ethanol solution of the title compounds at room temperature over 48 h.

### Refinement

All H atoms were placed geometrically with C–H = 0.95 (aromatic H), 0.99 (methylene H) and were refined using a riding model with *U*_iso_(H) = 1.2*U*_eq_(C).

### Crystal data

**Table:**

C_12_H_7_N_3_OS	*F*(000) = 496
*M**_r_* = 241.28	*D*_x_ = 1.470 Mg m^−^^3^
Monoclinic, *C**c*	Mo *K*α radiation, λ = 0.71075 Å
Hall symbol: C -2yc	Cell parameters from 1540 reflections
*a* = 16.979 (9) Å	θ = 2.5–29.9°
*b* = 9.407 (5) Å	µ = 0.28 mm^−^^1^
*c* = 7.034 (4) Å	*T* = 100 K
β = 103.927 (11)°	Blade, pale brown
*V* = 1090.5 (10) Å^3^	0.24 × 0.12 × 0.04 mm
*Z* = 4	

### Data collection

**Table:**

Rigaku AFC12 (Right) diffractometer	1986 independent reflections
Radiation source: Rotating Anode	1955 reflections with *I* > 2σ(*I*)
Detector resolution: 28.5714 pixels mm^-1^	*R*_int_ = 0.015
profile data from ω–scans	θ_max_ = 27.5°, θ_min_ = 3.7°
Absorption correction: multi-scan (*CrystalClear-SM Expert*; Rigaku, 2012)	*h* = −21→21
*T*_min_ = 0.944, *T*_max_ = 1.000	*k* = −12→11
3632 measured reflections	*l* = −9→9

### Refinement

**Table:**

Refinement on *F*^2^	Hydrogen site location: inferred from neighbouring sites
Least-squares matrix: full	H-atom parameters constrained
*R*[*F*^2^ > 2σ(*F*^2^)] = 0.022	W = 1/[Σ^2^(*F*_o_^2^) + (0.0377*P*)^2^ + 0.4177*P*] where *P* = (*F*_o_^2^ + 2*F*_c_^2^)/3
*wR*(*F*^2^) = 0.061	(Δ/σ)_max_ < 0.001
*S* = 1.08	Δρ_max_ = 0.19 e Å^−^^3^
1986 reflections	Δρ_min_ = −0.18 e Å^−^^3^
154 parameters	Absolute structure: Flack *x* parameter determined using 718 quotients [(I+)-(I-)]/[(I+)+(I-)] (Parsons & Flack, 2004)
2 restraints	Flack parameter: 0.03 (3)

### Special details

**Table:**

Geometry. Bond distances, angles *etc*. have been calculated using the rounded fractional coordinates. All su's are estimated from the variances of the (full) variance-covariance matrix. The cell e.s.d.'s are taken into account in the estimation of distances, angles and torsion angles
Refinement. Refinement on *F*^2^ for ALL reflections except those flagged by the user for potential systematic errors. Weighted *R*-factors *wR* and all goodnesses of fit *S* are based on *F*^2^, conventional *R*-factors *R* are based on *F*, with *F* set to zero for negative *F*^2^. The observed criterion of *F*^2^ > σ(*F*^2^) is used only for calculating -*R*-factor-obs *etc*. and is not relevant to the choice of reflections for refinement. *R*-factors based on *F*^2^ are statistically about twice as large as those based on *F*, and *R*-factors based on ALL data will be even larger.

### Fractional atomic coordinates and isotropic or equivalent isotropic displacement parameters (Å^2^)

**Table:**

	*x*	*y*	*z*	*U*_iso_*/*U*_eq_	
S1	0.14621 (4)	0.73643 (4)	0.22599 (6)	0.0152 (1)	
O1	0.23305 (9)	0.57530 (15)	−0.1843 (2)	0.0179 (4)	
N1	0.27769 (10)	0.68589 (17)	0.1115 (2)	0.0120 (4)	
N2	0.45449 (12)	0.8278 (2)	0.5329 (3)	0.0210 (6)	
N3	0.22781 (12)	0.91044 (19)	0.6832 (3)	0.0209 (5)	
C1	0.25143 (14)	0.74325 (18)	0.2647 (3)	0.0122 (5)	
C2	0.21787 (12)	0.6278 (2)	−0.0407 (3)	0.0138 (5)	
C3	0.13519 (12)	0.6388 (2)	0.0001 (3)	0.0162 (6)	
C4	0.29897 (12)	0.8016 (2)	0.4321 (3)	0.0135 (5)	
C5	0.38554 (13)	0.8131 (2)	0.4803 (3)	0.0146 (5)	
C6	0.25922 (12)	0.8615 (2)	0.5710 (3)	0.0149 (5)	
C7	0.36194 (12)	0.6787 (2)	0.1046 (3)	0.0128 (5)	
C8	0.40498 (13)	0.5558 (2)	0.1676 (3)	0.0157 (6)	
C9	0.48583 (13)	0.5491 (2)	0.1579 (3)	0.0205 (6)	
C10	0.52117 (14)	0.6634 (3)	0.0838 (3)	0.0218 (6)	
C11	0.47629 (14)	0.7848 (3)	0.0200 (3)	0.0207 (6)	
C12	0.39603 (13)	0.7932 (2)	0.0298 (3)	0.0159 (6)	
H3A	0.11320	0.54270	0.01290	0.0190*	
H3B	0.09750	0.68900	−0.10840	0.0190*	
H8	0.38000	0.47790	0.21620	0.0190*	
H9	0.51700	0.46640	0.20200	0.0250*	
H10	0.57630	0.65810	0.07680	0.0260*	
H11	0.50070	0.86240	−0.03050	0.0250*	
H12	0.36490	0.87600	−0.01390	0.0190*	

### Atomic displacement parameters (Å^2^)

**Table:**

	*U*^11^	*U*^22^	*U*^33^	*U*^12^	*U*^13^	*U*^23^
S1	0.0091 (2)	0.0180 (2)	0.0189 (2)	0.0011 (2)	0.0041 (2)	−0.0031 (2)
O1	0.0177 (7)	0.0190 (7)	0.0177 (8)	−0.0020 (6)	0.0058 (6)	−0.0049 (6)
N1	0.0093 (8)	0.0128 (7)	0.0142 (8)	0.0004 (6)	0.0032 (6)	−0.0010 (6)
N2	0.0165 (10)	0.0269 (9)	0.0201 (10)	−0.0019 (8)	0.0055 (8)	−0.0041 (7)
N3	0.0170 (9)	0.0251 (9)	0.0210 (10)	−0.0010 (8)	0.0052 (8)	−0.0074 (8)
C1	0.0116 (9)	0.0108 (8)	0.0149 (10)	0.0013 (7)	0.0044 (8)	0.0018 (7)
C2	0.0126 (9)	0.0115 (8)	0.0166 (9)	0.0007 (7)	0.0024 (8)	0.0016 (7)
C3	0.0117 (10)	0.0203 (9)	0.0157 (10)	−0.0015 (8)	0.0018 (8)	−0.0019 (8)
C4	0.0113 (10)	0.0132 (8)	0.0161 (10)	0.0013 (7)	0.0034 (8)	−0.0010 (7)
C5	0.0159 (11)	0.0142 (8)	0.0143 (9)	−0.0007 (8)	0.0046 (8)	−0.0016 (7)
C6	0.0109 (9)	0.0150 (9)	0.0173 (10)	−0.0017 (8)	0.0002 (8)	−0.0018 (7)
C7	0.0097 (9)	0.0167 (8)	0.0126 (9)	−0.0010 (7)	0.0040 (8)	−0.0034 (7)
C8	0.0158 (10)	0.0183 (9)	0.0136 (10)	0.0013 (8)	0.0046 (8)	−0.0003 (7)
C9	0.0152 (10)	0.0284 (10)	0.0172 (10)	0.0070 (9)	0.0026 (8)	−0.0017 (8)
C10	0.0108 (9)	0.0389 (12)	0.0162 (10)	0.0003 (9)	0.0044 (8)	−0.0052 (9)
C11	0.0158 (11)	0.0299 (11)	0.0175 (11)	−0.0080 (9)	0.0064 (9)	−0.0001 (9)
C12	0.0141 (10)	0.0176 (9)	0.0145 (10)	−0.0015 (8)	0.0008 (9)	0.0005 (7)

### Geometric parameters (Å, º)

**Table:**

S1—C1	1.742 (3)	C7—C12	1.385 (3)
S1—C3	1.806 (2)	C8—C9	1.392 (3)
O1—C2	1.207 (3)	C9—C10	1.392 (3)
N1—C1	1.372 (3)	C10—C11	1.386 (4)
N1—C2	1.398 (3)	C11—C12	1.383 (3)
N1—C7	1.445 (3)	C3—H3A	0.9900
N2—C5	1.148 (3)	C3—H3B	0.9900
N3—C6	1.150 (3)	C8—H8	0.9500
C1—C4	1.372 (3)	C9—H9	0.9500
C2—C3	1.503 (3)	C10—H10	0.9500
C4—C5	1.431 (3)	C11—H11	0.9500
C4—C6	1.430 (3)	C12—H12	0.9500
C7—C8	1.382 (3)		
			
C1—S1—C3	92.38 (10)	C8—C9—C10	120.15 (19)
C1—N1—C2	116.22 (18)	C9—C10—C11	120.3 (2)
C1—N1—C7	123.81 (17)	C10—C11—C12	120.2 (2)
C2—N1—C7	119.93 (16)	C7—C12—C11	118.8 (2)
S1—C1—N1	112.19 (15)	S1—C3—H3A	110.00
S1—C1—C4	121.22 (17)	S1—C3—H3B	110.00
N1—C1—C4	126.6 (2)	C2—C3—H3A	110.00
O1—C2—N1	122.62 (19)	C2—C3—H3B	110.00
O1—C2—C3	125.85 (19)	H3A—C3—H3B	109.00
N1—C2—C3	111.53 (17)	C7—C8—H8	121.00
S1—C3—C2	107.42 (14)	C9—C8—H8	121.00
C1—C4—C5	126.3 (2)	C8—C9—H9	120.00
C1—C4—C6	117.9 (2)	C10—C9—H9	120.00
C5—C4—C6	115.80 (18)	C9—C10—H10	120.00
N2—C5—C4	174.4 (2)	C11—C10—H10	120.00
N3—C6—C4	179.4 (2)	C10—C11—H11	120.00
N1—C7—C8	118.67 (18)	C12—C11—H11	120.00
N1—C7—C12	118.95 (18)	C7—C12—H12	121.00
C8—C7—C12	122.4 (2)	C11—C12—H12	121.00
C7—C8—C9	118.27 (18)		
			
C3—S1—C1—N1	4.42 (14)	N1—C1—C4—C5	−0.5 (3)
C3—S1—C1—C4	−175.95 (16)	S1—C1—C4—C6	−2.2 (3)
C1—S1—C3—C2	−4.78 (14)	S1—C1—C4—C5	179.94 (15)
C7—N1—C1—S1	179.56 (14)	N1—C1—C4—C6	177.33 (18)
C2—N1—C1—C4	177.70 (18)	N1—C2—C3—S1	4.19 (19)
C7—N1—C1—C4	0.0 (3)	O1—C2—C3—S1	−176.30 (17)
C7—N1—C2—C3	176.70 (16)	N1—C7—C8—C9	179.13 (17)
C1—N1—C2—O1	179.35 (18)	C12—C7—C8—C9	1.1 (3)
C7—N1—C2—O1	−2.8 (3)	N1—C7—C12—C11	−178.78 (18)
C2—N1—C1—S1	−2.7 (2)	C8—C7—C12—C11	−0.8 (3)
C2—N1—C7—C12	94.9 (2)	C7—C8—C9—C10	−0.9 (3)
C1—N1—C7—C8	94.5 (2)	C8—C9—C10—C11	0.3 (3)
C1—N1—C2—C3	−1.1 (2)	C9—C10—C11—C12	0.1 (3)
C1—N1—C7—C12	−87.5 (2)	C10—C11—C12—C7	0.2 (3)
C2—N1—C7—C8	−83.2 (2)		

### Hydrogen-bond geometry (Å, º)

Cg2 is the centroid of the C7–C12 phenyl ring.

**Table:**

*D*—H···*A*	*D*—H	H···*A*	*D*···*A*	*D*—H···*A*
C10—H10···N3^i^	0.95	2.58	3.479 (4)	157
C8—H8···*Cg*2^ii^	0.95	2.96	3.610 (3)	127

Symmetry codes: (i) *x*+1/2, −*y*+3/2, *z*−1/2; (ii) *x*, −*y*+1, *z*+1/2.
